# P168: Leadership styles of ward head nurses and implementation success – a qualitative inquiry in the framework of a mixed-method study on hand hygiene promotion through patient involvement

**DOI:** 10.1186/2047-2994-2-S1-P168

**Published:** 2013-06-20

**Authors:** S Touveneau, L Clack, C Ginet, A Stewardson, M Schindler, M Bourrier, D Pittet, H Sax

**Affiliations:** 1 Infection Control Program, University of Geneva Hospitals, Geneva 14, Switzerland; 2 Division of Infectious Diseases and Hospital Epidemiology, University Hospital Zurich, CH-8091 Zurich, Switzerland; 3 Faculté des Sciences Economiques et Sociales, University of Geneva, Geneva, Switzerland

## Introduction

To actively involve patients in hand hygiene promotion is now widely advocated but many institutions find this challenging.

## Objectives

We sought to understand the role of the ward head nurses (HN) in the implementation process of a patient participation (PP) module in a randomized controlled effectiveness trial on hand hygiene promotion.

## Methods

A case study was conducted using a mixed deductive and inductive approach based on the grounded theory (Glaser & Strauss, 1967). During the active implementation phase we interviewed head nurses of the 19 study wards quarterly regarding their attitude towards PP, their promotional engagement, their knowledge of the ward’s implementation progress, and their account of facilitators and barriers.

## Results

The head nurses’ intrinsic leadership characteristics and their ‘perception of PP’ emerged as key elements for successful implementation. Plotted as a matrix, they formed winning or losing combinations in regards to implementation success. ‘Promotional activity’ types ‘refusing’ and ‘pretending’ were unsuccessful, while ‘proactive managing’ and ‘innovation’ were winning combinations. Additionally, we saw ‘perception of PP’ evolve over the course of the study as a function of prior implementation experience and the hierarchical and team support they received. Patients very rarely reminded healthcare workers (HCW) to do hand hygiene, but awareness of the importance of hand hygiene rose amongst both patients and HCW.

## Conclusion

Intrinsic leadership style in the organization’s mid-level managers and attitude towards the intervention were the most important predictors for successful implementation of this very challenging task, and organizational context also played an important modifying role. We saw unanticipated evolution of attitudes and activities throughout the study period. Thus, we suggest involving effective leaders and providing them with continuous organizational support to ensure implementation success.

## Disclosure of interest

None declared

